# Toward a Procedure to Study Rule-Governed Choice: Preliminary Data

**DOI:** 10.1007/s40616-024-00206-6

**Published:** 2024-05-28

**Authors:** David Ruiz Méndez

**Affiliations:** Laboratorio de Análisis de la Conducta, Facultad de Estudios Superiores Iztacala, Universidad Nacional Autónoma de México, de los Barrios Ave. Number 1, Los Reyes Ixtacala, Tlalnepantla, , Estado de México C.P. 54090 México

**Keywords:** Choice, Rules, Rule-following, Rate of reinforcement, Humans

## Abstract

The aim of this study was to model a situation that induced choice between following two incompatible rules, each associated with a different rate of reinforcement. In Experiment 1, eight undergraduate students were exposed to a two-component multiple schedule (training). In each component, there was a concurrent variable interval (VI)–extinction (EXT) schedule. Participants were given two rules that instructed them to respond to the VI alternative in the presence of different discriminative stimuli. The side of the VI schedule changed in each component and offered a different reinforcer rate according to the discriminative stimuli in the operation. When both discriminative stimuli were concurrently presented (test), participants favored the alternative previously instructed by the rule, which was associated with the greatest reinforcer rate, whereas indifference was observed in the absence of discriminative stimuli. Experiment 2 tested the effects of reinforcement rate using the same procedure without providing rules. During training, participants gradually developed a preference for the VI alternatives. In the choice test phase, participants favored the alternative associated with the stimuli with the highest reinforcer rate when both discriminative stimuli were present. Unsystematic preference was observed in the absence of discriminative stimuli. Two alternative explanations were provided for the findings.

Rules and rule-governed behavior are central topics in the experimental analysis of human behavior (Fienup, 2019; Hayes et al., 1986; Petursdottir & Devine, 2017). Rule-governed behavior has been defined as behavior under the control of a rule (Baron & Galizio, 1983; Baum, 2016; Catania et al., 1989; Cerutti, 1989). As an example, imagine someone who regularly avoids driving a certain route because she was warned about the damaged road, even though that person has never driven through that road before. In contrast, contingency-shaped behavior is directly maintained by the consequences it produces without the need of verbal mediation (Catania et al., 1989; Hayes et al., 1989; Skinner, 1974). To exemplify, imagine another person who also avoids the same route but for a different reason: following it led to a flat tire. There are many theoretical controversies around the concept of rules and verbal behavior in general (Barnes-Holmes et al., 2002; Fryling, 2013; Gross & Fox, 2009; Shimoff & Catania, 1998). However, here, *rules* are viewed as verbal function-altering stimuli (Schlinger, 1990; Schlinger & Blakely, 1987; Vaughan, 1989).

Rule following is a common behavior in our everyday lives (Baum, 1995, 2016; Skinner, 1957, 1974, 1984). One of the many reasons is that rules can promote the rapid acquisition of novel and adaptative behavior (Ayllon & Azrin, 1964; Neef et al., 2004; Tiger & Hanley, 2004). One does not need to directly experience the consequences of ignoring a red traffic light (car crash) or eating certain poisonous plants (illness or death) because many community-provided rules are designed to preserve the safety of the follower. Thus, even when rules are also linked to undesirable effects (Catania et al., 1989; McAuliffe et al., 2014; Shimoff et al., 1981), they play a role in human activities and must be included in any naturalistic and evolutionary explanation of human behavior (Baum, 1995).

Given that rules can take many forms (advice, instructions, orders, and morals; Baum, 2016), it is not hard to think of situations in which incompatible rules might concurrently compete for control. For example, consider a child whose mother told her, “When you get to school, please use the scarf we gave you.” However, during the ride to school, a friend tells her, “If you want to sit with us in school, do not wear that silly scarf.” In this situation, both rules *conflict* with each other because the contingencies specified by the rules are incompatible. Thus, this situation involves a choice between following two different rules. To continue with the example, imagine that upon arriving at school the child decides to wear the scarf over sitting with the other children. Does her preference reflect the relative effect that each of the rules had over her behavior in that situation?

To understand this choice situation, we must start by acknowledging that rule-following is operant behavior. Experimental and theoretical work has suggested that rules acquire their function-altering effects over responding through differential reinforcement (Buskist & Miller, 1986; DeGrandpre & Buskist, 1991; Galizio, 1979; Hackenberg & Joker, 1994; Newman et al., 1995; Schmitt, 1998; Sundberg, 2016). Therefore, if the effect of rules on behavior is determined by the consequences associated with following them, then, in choice situations where the alternatives involve following incompatible rules, preference might be determined by the same variables governing operant choice. Research on choice situations show temporally extended variables such as reinforcer rate determine choice allocation for both humans (Baum, 1975; Horne & Lowe, 1993; Krägeloh et al., 2010; Pierce & Epling, 1983; Ruiz et al., 2021) and non-human organisms (Baum, 1973, 1974, 2010; Davison & McCarthy, 1988; Grace & Hucks, 2013). Therefore, if rule-following behavior is conceptualized as operant behavior, then a choice between rules might be determined by the reinforcer rate of each rule. To provide evidence for this claim, an experiment must demonstrate that by holding the consequence type constant and manipulating the reinforcer rate associated with following two conflicting rules, behavior conforming to what was stated by the “richer rule” (the one with the highest correlation with reinforcement) should prevail when choice is induced.

The situation above can be modeled in a laboratory experiment using *instructed discrimination* (Cerutti, 1989). In this type of rule-following episode, instructional stimuli (rules) enhance the stimulus control between a property of a stimulus and an instructed response. For example, if someone tells you, “When you hear the bell ring, open the door,” the rule alters the function of a stimulus (the sound of the bell) in relation to operant responding (opening the door). This makes the whole discriminated operant the defining component of the rule-following episode. Using instructed discrimination, one could test the relative function-altering effects of two conflicting rules as follows: first, different rules could be created, each specifying incompatible patterns of behavior according to the presence of different discriminative stimuli; second, differential histories of reinforcement associated with each rule could be arranged. This could be done by providing differential reinforcement using a multiple schedule in which a different rule is followed depending on the component in operation (training); third, evidence for rule-following behavior should be provided during training so that, fourth, the choice between the rules could be induced by concurrently presenting both discriminative stimuli associated with each of the rules in a probe test.

The aim of this research was to model a situation to study the way two conflicting rules, each associated with different rates of reinforcement, competed for control in a concurrent-choice procedure. The experimental preparation involved responding to two alternatives (a left or a right button) in the presence of two different discriminative stimuli (a flashing circle that changed colors from red to blue). Two rules were designed. Each rule described the behavior that a participant had to emit (see Matthews et al., 1985) in the presence of two different discriminative stimuli (e.g., when you see the red circle, press the left button). Given that each rule instructed responding *exclusively* to one of two available alternatives (either to the left or to the right) in the presence of the different discriminative stimuli (red/blue), the instructed contingencies were incompatible if both discriminative stimuli were present. During training, each rule was reinforced separately using a multiple schedule of reinforcement. Different reinforcer rates were arranged for following the two different rules. Following training, participants completed a test that assessed if they could correctly recall the contingencies instructed by the two rules by completing different sentences. If they were successful on their first try without any errors, the final step consisted of probing their behavior by presenting them with both discriminative stimuli at the same time. If rule-following is determined by the correlation between following a rule and receiving reinforcement, then when both rules can be followed (both stimuli are present), behavior based on the rule correlated with the highest reinforcer rate should prevail.

## Experiment 1

### Method

#### Participants

Eight undergraduate students, three men and five women, between 18 and 20 years old were recruited from a class of General Psychology at a University in the State of México. Given the restrictions of the university to access physical infrastructure owing to the COVID-19 pandemic, participants performed the experiment from their homes using Zoom® videoconferencing software. The information from the experiment, as well as the procedure to participate in the experiment, were posted on the institutional university webpage.

To participate in the experiment, students had to complete an online questionnaire that requested information about their academic background and any visual perception disabilities. This information was required to determine if participants could participate in the experiment (see below). They also had to submit a screenshot of the results of an online internet velocity test applied to their internet connection (https://www.speedtest.net/). Inclusion criteria to participate in the experiment were being a first-year undergraduate student enrolled in the Psychology program at the university. This was done to avoid participants knowing about behavior analysis or theory regarding schedules of reinforcement. Exclusion criteria were being diagnosed with color vision deficiency and/or reporting an internet connection lower than 10 Mbps (download) – 10 Mbps (upload). Participants provided written informed consent and were told that their participation was voluntary and that they could withdraw from the experiment at any time without any consequence. The research protocol and the informed consent form were reviewed and approved by the Ethics Committee of the university.

#### Apparatus

All experimental sessions were conducted online with an HP 240G4 Notebook PC running Windows 10. The experimental task was presented to participants using Zoom® Videoconference software via the “Remote control” function. This function allowed participants to control the experimenter’s computer remotely and interact with the task in real time. The experimenter used an internet connection of 50 Mbps (download) – 50 Mbps (upload) with a ping of 25 ms for the experiment. The programming of the experimental task and the data collection system were accomplished by the author using Visual Studio 2019® for Windows.

#### Setting

All participants performed the experiment from their homes using Zoom® videoconferencing software. When participants entered the Zoom session, they had to demonstrate that they were alone in a quiet room by showing the experimenter the room they were in through their cameras. All participants met this requirement. Participants’ microphone and camera were requested to be turned on during the whole session. None of the participants experienced technical problems with their microphones or cameras. The experimenter had the camera turned on while the experiment took place but left the room after reading the instructions to eliminate the effects of his presence. The angle of the experimenter’s camera was arranged so that participants could see the experimenter leaving or entering the room. When the session finished, the task produced a sound that alerted the experimenter, so he could enter the room and debrief the participant. The experiment was conducted in a single session and lasted for about an hour for all participants. At the end of the experiment, the computer program showed the participant how much money they had earned. Participants earned an average of 90 pesos (5 US dollars) by the end of the session. Participants received money through an electronic bank deposit at the end of the session. Then, the experimenter debriefed and dismissed participants.

#### Procedure

**Fig. 1:**
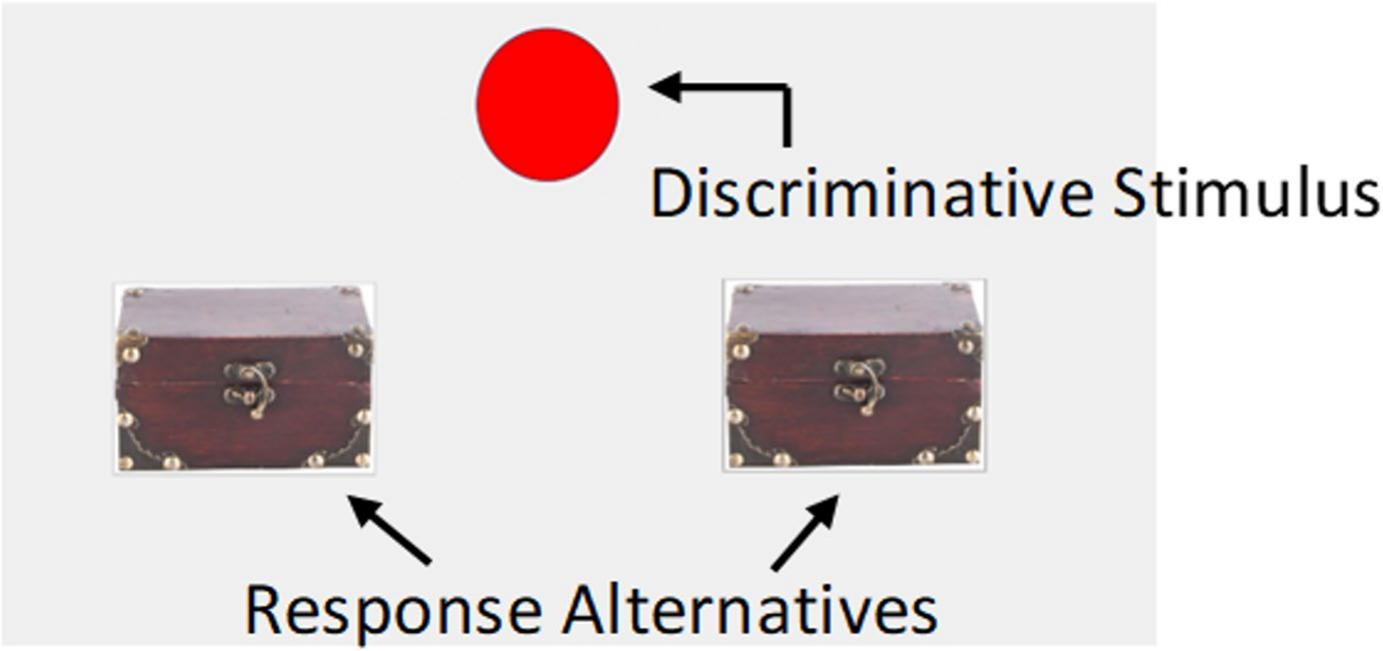
An example of the screen of the experimental task during a component presentation

The experiment consisted of three experimental phases: *training, the rule-test phase, and the choice-test phase*. Experimental phases were presented in that order for all participants in all conditions. The *training phase* was designed to create a differential history of reinforcement to follow two rules in a clearly discriminable context that precisely signaled which rule had to be followed. During this phase, the experimental task involved a game where participants had to press one of two buttons, located on each side of the screen (Fig. 1). A 4.5 cm circle at the center top of the screen functioned as a discriminative stimulus (Fig. 1). The color of this circle changed from red to blue depending on the component in operation. The response alternatives were two identical 471 x 358 pixel buttons located on each side of the screen. Each button had an image of a chest (Fig. 1). Hereafter, the buttons will be referred to as the alternatives.

##### Training Phase

The training phase consisted of the presentation of three blocks of six cycles of a two-component multiple schedule. Each six-cycle block was separated by a 30 s resting period. A cycle consisted of the presentation of the two components of the multiple schedule, each separated by a 10 s inter-component interval. The discriminative stimulus in each of the components was either a red or a blue circle. During a component, the colored circle constantly flashed at a rate of one flash per 235 ms to enhance its salience. Each of the components of the multiple schedule was 60 s, making 140 s the total duration of a cycle. Each component presentation was considered as an individual observation period. The inter-component interval was implemented to reduce carry over from previous components (Baron & Perone, 1998; Davison & Baum, 2002). The order of component presentation within a cycle was randomly determined at the cycle onset by the program.

During a component, participants could freely respond to the two response alternatives located on each side of the screen (Fig. 1). The alternatives were governed by two concurrent variable interval–extinction (VI–EXT) schedules. The operation of each concurrent schedule depended on the component in operation (Table 1). The two concurrent schedules had different VI schedules: a VI 60 s and a VI 10 s. The VI schedule of each concurrent schedule operated on either the left or the right alternative depending on the component in operation. The rules always instructed participants to respond to the VI in the presence of the different discriminative stimuli (see Table 1), making rule-compatible behavior the only behavior associated with reinforcement within a component. Schedule parameters were counterbalanced across participants in relation to the side of the VI and the color of the discriminative stimulus (see Table 1).

**Table 1:** Experimental parameters for each participant in Experiment 1

Participant	Discriminative stimuli	Component schedule	Rules provided
GP101	Red	VI-10 s – EXT	When the circle is red, press the left chest.
	Blue	EXT – VI-60 s	When the circle is blue, press the right chest.
GP201	Blue	VI-10 s – EXT	When the circle is blue, press the left chest.
	Red	EXT – VI-60 s	When the circle is red, press the right chest.
GP301	Red	EXT – VI-10 s	When the circle is red, press the right chest.
	Blue	VI-60 s – EXT	When the circle is blue, press the left chest.
GP401	Blue	EXT – VI-10 s	When the circle is blue, press the right chest.
	Red	VI-60 s – EXT	When the circle is red, press the left chest.
GP102	Red	VI-10 s – EXT	When the circle is red, press the left chest.
	Blue	EXT – VI-60 s	When the circle is blue, press the right chest.
GP202	Blue	VI-10 s – EXT	When the circle is blue, press the left chest.
	Red	EXT – VI-60 s	When the circle is red, press the right chest.
GP302	Red	EXT – VI-10 s	When the circle is red, press the right chest.
	Blue	VI-60 s – EXT	When the circle is blue, press the left chest.
GP402	Blue	EXT – VI-10 s	When the circle is blue, press the right chest.
	Red	VI-60 s – EXT	When the circle is red, press the left chest.

*Note:* Component schedules were concurrent schedules of reinforcement. For example, “VI-10 s – EXT” means that the variable interval schedule operated on the left alternative and the extinction schedule operated on the right alternative

Each VI schedule contained 20 intervals generated according to the method proposed by Fleshler and Hoffman (1962). This method allowed the creation of individual intervals that followed an exponential memoryless function, resulting in constant-probability VI schedules. Each time a component started, a list of intervals was generated for the VI schedule. An interval from that list was randomly assigned to the operandum with the VI. A reinforcer consisted of the delivery of a point on the screen equivalent to 0.7 cents of a Mexican peso (0.034 US dollars at the time of the experiment). Each time a reinforcer was delivered, the computer program selected a new interval without replacement from the list and was assigned to the operandum that delivered the reinforcer. Having different concurrent VI–EXT schedules programmed for each of the components in combination with the rules guaranteed a marked difference in the programmed rate of reinforcement associated with following each of the rules. On the other hand, rule-incompatible behavior was put on extinction in both components, so that this behavior could not be a confounding factor later in the choice test.

During each of the components, a 3 s changeover delay (COD) was in effect. This contingency was added to discourage rapid alternation between the alternatives during a component. The COD consisted of the removal of the operanda from the screen each time a participant switched between alternatives, so no responses could be emitted during this 3 s period. While the operanda was removed from the screen, a 9 x 11.5 cm rectangle appeared with the text “Wait.” The rectangle was displayed for 3 s after which the response alternatives reappeared. The selection of the first response during component onset was recorded so that the contingency could be activated with the first response to the other alternative. Each time a COD occurred, the first response to the switched alternative was reinforced while a response to the pre-COD alternative produced another COD.

Instructions (described in more detail later) consisted of two rules that directed participants to always respond to the VI alternative according to the different discriminative stimuli (Table 1). Given the different programmed reinforcer rates associated with each VI schedule operating for each of the components, responding in accordance with the rules produced a globally rich and a globally lean component, hereafter referred to as the *Rich* and *Lean* components. This arrangement had the following advantages: First, it presented a clearly discriminable context (red or blue circle) that precisely signaled which of the two rules had to be followed while manipulating the reinforcer rate associated with following each of the rules. Second, the difference in the rates of reinforcement associated with following each of the rules was pronounced (six to one programed reinforcers per minute, on average), increasing the probability of contingency discrimination. Third, following the rule in the Lean component sometimes resulted in no reinforcement when an interval sampled from the list of possible intervals *(M* = 60 s) was greater than the duration of the component (60 s). This effectively turned the actual program into a concurrent EXT–EXT schedule. This schedule was programmed for a component randomly in six of the 18 components of the training phase. The non-reinforced components were used as additional tests to see if rule-consistent behavior was observed, even when it was not reinforced.

To advance to the next phase, 95% or more of participants’ responses in the last six observation periods had to allocate to the instructed alternative in both components. If both criteria were not met by a participant, then their data were excluded from the study, since component discrimination in accordance with the rules was a fundamental prerequisite for the next phase. No data were excluded, since all participants met the criterion.

##### Rule-test Phase

This phase tested if participants could recall the rules provided at the beginning of the task before the last phase started. In this phase, participants were presented with four incomplete sentences that described the instructional content stated by the rules. Participants had to complete the four sentences without any errors by writing the word that correctly completed each sentence in accordance with the rules. If participants failed, then rule compliance during the first phase was questioned, indicating that no further testing could be conducted.

There were two types of sentences. The first sentence group was called “color sentences.” An example of a sentence of this group was, “When the circle is _ [text input box]_ , the [*left/right*] treasure chest must be pressed.” Participants had to write in the text input box *the color of the circle* according to the content of the sentence. The second sentence group was called “side sentences.” When a sentence of this group appeared, participants had to write in the text input box the *side of the alternative* according to the content of the sentence. The structure of this type of sentence was, “When the circle is *red/blue*, the _ [text input box] treasure chest must be pressed.” The two sides and the two colors were tested. The correct answer depended on the specific rules each participant was given (Table 1).

To check if their answer was correct, participants had to validate their answer by writing their answers inside the input box and then clicking on a button at the bottom of the screen labeled “validate.” The following text appeared at the top of all sentences, “Always answer using lowercase letters and omit any spaces between letters.” This was programmed to prevent participants from making any errors owing to the use of spaces or uppercase letters, since exact responses with lowercase letters were required. Once participants validated their answers, feedback in the form of a window showing the legend “Correct” or “Incorrect” was provided. Additionally, each time the “validate” button was clicked and feedback was provided, the original response to that sentence was recorded and could not be changed. After that, the next sentence appeared, so if participants provided an incorrect or inaccurate response (spaces or uppercase letters), then it counted as an error.

The four sentences were presented in random order to participants to avoid any bias toward one of the rules. The order was decided at the phase onset by the program. The presentation of the four sentences was considered a cycle. Participants had to complete all four sentences correctly within the first cycle (100% of correct responses) to advance to the last experimental phase. If they failed, the experiment ended, and their data were excluded. All participants completed this test successfully within their first cycle without any errors. As a result, no participants were excluded from the experiment at the end of this phase.

##### Choice-test phase

The last phase was termed the *choice-test phase* and was the central part of the experiment. It was designed to test the relative effects of the two incompatible rules by inducing choice. In this phase, a four-component multiple schedule was in operation. In addition to presenting the Rich and Lean components, two more components were added: a component termed “Both Stimuli” (BS) and a component termed “No Stimulus” (NS). The test phase consisted of the presentation of two cycles of the four-component multiple schedule. During the BS component, the component stimulus was the same previously used circle but changed constantly from red to blue. The time interval for a color to be displayed before it changed was 400 ms. This parameter was arranged in combination with the COD as to make it impossible for participants to rapidly alternate between buttons and keep up with the change in colors. Whether the circle was red or blue at the beginning of this component was randomly determined at the component onset as well as the order of the components. In the NS condition, the circle at the center top of the screen was removed. Both the BS and NS components were 60 s and no reinforcement was programed for both alternatives (Extinction).

##### Instructions

General instructions for all participants were as follows:

Welcome to “find the treasure.”

You are going to see two treasure chests on the screen. One on the left and one on the right. You must click on them to try to open them and get money. Each time you open a treasure chest, you will receive 0.7 cents of a peso (0.05 US dollars).

You will find a circle that flashes at the top of the screen. This circle constantly changes its color. Pay attention to the following indications:

--------------------------------------------------------------------------------------------

--------------------------------------------------------------------------------------------

Try to earn as much money as possible. Sometimes, questions will appear on the screen. Always answer using lowercase letters and omit any spaces between letters.

At the end of the experiment, the amount of money you gained will appear on the screen. If you do not have any doubts, click on the button “Continue.”

The two “indications” that appeared in the place of the dashed lines were the two performance rules that stated which button to press in the presence of a different color according to the parameter combination each participant had (see Table 1). Once instructions appeared, it was randomly decided which one of the two rules was in the first row.

#### Dependent Variables and Data Analysis

The experimenter first calculated the total emitted responses to each of the alternatives during each component in Experiment 1 and 2. With the obtained response counts, preference measures were calculated in the form of response proportions to each of the alternatives. The primary dependent variable was the percentage of responses allocated to the instructed alternative within a component. In the training phase, the experimenter estimated this percentage as a function of each successive observation period. A value closer to 100% indicated preference for the instructed alternative, a percentage of 50% indicated indifference, and a percentage closer to 0% indicated preference for the uninstructed alternative. This data representation strategy provided a common quantitative scale to assess how much participants’ behavior conformed to what was stated by the rules. Additionally, mean response counts to each of the alternatives (considering the last six observation periods in accordance with our stability criterion) in each of the components were also reported.

Similar response percentages were estimated in the choice-test phase. For the Rich and Lean components, the percentage of responses allocated to the instructed and uninstructed alternatives was used. For the BS and NS components, the experimenter estimated the percentage of responses allocated to the alternative that was instructed by the rule applicable to the Rich component (Rich) and the percentage of responses to the alternative that was instructed by the rule applicable to the Lean component (Lean). Since there were only two alternatives in the task (left–right), the two rules instructed incompatible behavior when both discriminative stimuli were present. Therefore, if participants correctly recalled the two rules before the choice-test phase, this percentage was interpreted as a potential measure of the effect each rule exerted over participants’ behavior when both could be followed (BS component). For example, if the percentage of responses to the Rich alternative in the BS and NS components was higher than for the Lean alternative, then this could indicate greater control by the rule associated with the highest reinforcer rate if rule-following behavior occurred.

Given that the interest in the training and the choice-test phase was to assess the differences in response allocation between the alternatives during a component, multiple pairwise comparisons using two-tailed Fisher–Pitman exact permutation tests for related samples were conducted. This non-parametric permutation approach compares differences in means for dependent data (Hollander & Wolfe, 1999). Permutation tests build an empirical null distribution by rearranging data according to all possible permutations between the conditions (Belmonte & Yurgelun-Todd, 2001; Wilcox, 2017). This makes them useful for multiple comparisons because they do not make the common population assumptions and because potential irregularities in data are maintained when the distribution is estimated (Camargo et al., 2008; Cheverud, 2001). Each time a comparison was conducted, the experimenter reported the test statistic (*T*), the mean difference between the alternatives (*M*_*dif*_), and the associated *p* value. A *p* value less than 0.05 indicated a systematic difference in the means the conditions compared. All data analyses were performed using R (R Core Team, 2020) and Microsoft Excel. Permutation tests were carried out using the *twoSamplePermutationTestLocation* function available in the *EnvStats* package (Millard, 2013).

### Results

**Fig. 2:**
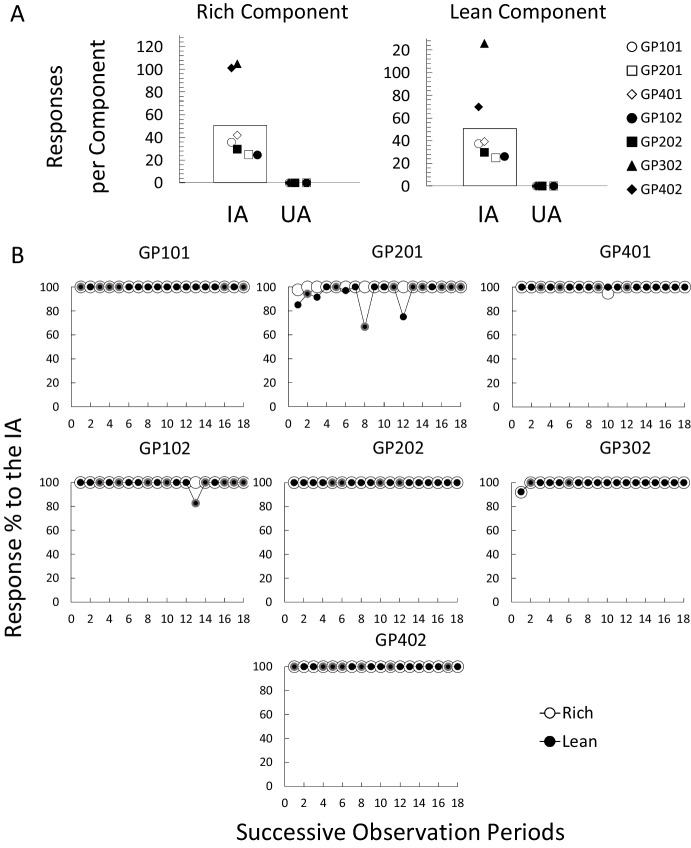
Choice analyses for the training phase. *Note*. Panel A. Response counts for each alternative in each of the components. Each data point represents the estimated response count for each participant in each of the alternatives, calculated as the mean data from the last six observation periods. Bars represent the median of all the data points. IA (Instructed alternative) and UA (Uninstructed alternative). Panel B. Percentage of responses to the instructed alternative as a function of successive observation periods. The black circles with a red border indicate observation periods where following the rule was not reinforced during the Lean component. Data from participant GP301 in the training phase was lost owing to a programming error during data analysis

Figure 2 shows the different analyses conducted for the training phase. Panels in section A show response counts for each alternative in each of the components. Each data point represents the estimated response count for each participant in each of the alternatives (instructed and uninstructed), calculated as the mean data from the last six observation periods in accordance with the stability criteria of this experiment. The bars represent the median value of all the data in each condition. Data from this phase for participant GP301 were lost owing to a programming error during the data analysis process and therefore are not shown. However, this participant met all the criteria to advance to the next phases, so her data were included in the following analyses. For all participants, at the end of the phase, all responses were always allocated to the instructed alternative regardless of the component (Rich *T* 363.23, *M*_*dif*_ 51.89, *p* = 0.01; Lean *T* 353.4, *M*_*dif*_ 50.48, *p* = 0.01). Moreover, levels of responding for the instructed alternative were about the same for most participants in the Rich (1st quartile 27.4*, Mdn* 35.83*,* 3rd quartile 71.6) and Lean (1st quartile 27.95*, Mdn* 37.5*,* 3rd quartile 54.6) alternatives at the end of the phase. The only exceptions were participants GP302 and GP402. Participant GP302 responded more to the instructed alternative during the Lean component, while GP402 responded more to the instructed alternative during the Rich component.

Panels in section B show the percentage of responses to the instructed alternative as a function of successive observation periods for each participant in the training phase. The empty circles represent preference to the instructed alternative in the Rich component and the filled circles represent preference to the instructed alternative in the Lean component. The filled circles with a gray border indicate observation periods during the Lean component when responding to the instructed alternative was not reinforced. Participants always favored the instructed alternative in both the Rich and Lean components. They also sampled the alternative under extinction, but this behavior was infrequent and was not exclusive of a component. For example, participant GP401 sampled the alternative under extinction in the Rich component, whereas participant GP102 did it in the Lean component. Participants GP201 and GP302 (in the first component) sampled the extinction alternative in both components. Most participants ceased to sample the uninstructed alternative during the last six observation periods, even when rule-following was not reinforced in the Lean component. The differences in reinforcer rate between components did not affect responding to the instructed alternative: preference for the instructed alternative was the same level for both components for most participants through the phase and for the last six observation periods (stability criteria).

**Fig. 3:**
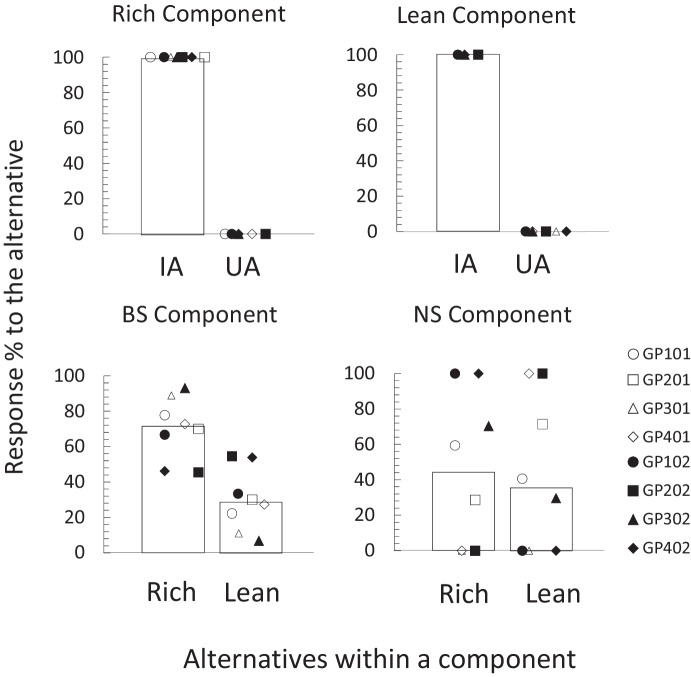
Choice analyses for the choice-test phase. *Note*. Percentage of responses to each alternative during each of the components of the choice-test phase. Bars represent the group median: IA (Instructed alternative), UA (Uninstructed Alternative), Rich (alternative instructed by the Rich Rule), Lean (alternative instructed by the Lean Rule)

All participants successfully completed the four sentences in the rule-test phase (100% of correct responses) on their first try. Therefore, all advanced to the last phase. Figure 3 shows the percentage of responses to each of the alternatives in each of the components during the choice-test phase. Given that in this phase each component was presented twice, in each component the experimenter pooled the two obtained response counts for each alternative, for each participant. Using the total counts obtained, the percentage of responses for each of the alternatives in all components was estimated. The top panels of Fig. 3 show the response percentages in the Rich and Lean components. As in the last observation periods during training, all participants allocated 100% of their responses to the instructed alternative (Rich *T* 800, *M*_*dif*_ 100, *p* < 0.01; Lean *T* 800, *M*_*dif*_ 100, *p* < 0.01).

The bottom panels of Fig. 3 show the percentage of responses to each of the alternatives in the test components. The bottom left panel shows the results for the BS component. Participants generally favored the alternative instructed by the Rich rule when both discriminative stimuli were present (*T* 321.54, *M*_*dif*_ 40.19, *p* = 0.03). Specifically, 6 of 8 participants allocated the majority of their responses to the alternative instructed by the Rich rule (Rich 1st quartile 66.7%*, Mdn*71.4%*,* 3rd quartile77.8%; Lean 1st quartile 22.2%*, Mdn* 28.6%*,* 3rd quartile 33.3%). The only exceptions were participants GP202 (Rich 45.5 %) and GP402 (Rich 46.2 %), who responded slightly more to the alternative instructed by the Lean rule. However, their preferences were closer to indifference.

Results in the NS component show a different picture. In this component, differences in responding between alternatives were unsystematic for individual participants (*T* 16.63, *M*_*dif*_ 2.07, *p* = 0.99; Rich 1st quartile 0%*, Mdn* 44%*,* 3rd quartile 70.4%; Lean 1st quartile 0%*, Mdn* 35.1%*,* 3rd quartile 71.4%). Four participants favored the alternative instructed by the Rich rule (GP101, GP102, GP302, and GP402) and three participants favored the alternative instructed by the Lean rule (GP201, GP401, and GP202). Participant GP301 was indifferent (Lean 0%, Rich 0 %).

### Discussion

The aim of this experiment was to test the competing effects of two different rules, each associated with different reinforcer rates and instructing incompatible behavior, in a choice situation. In the first phase (training), responding to the alternatives instructed by the rules was differentially reinforced. All participants immediately conformed to what was stated by each of the rules (Fig. 2 section B). Participants responded almost exclusively to the instructed alternative and this pattern of responding continued until the end of the phase. The response counts for the instructed alternatives were the same for both components, suggesting that the differential reinforcement programmed for following the two rules during this phase did not cause greater absolute responding to a particular alternative in a component. In the components where no reinforcement was provided for responding to the instructed alternative participants still responded to it. Some participants occasionally sampled the uninstructed alternative, but those episodes were brief, infrequent, and took place in both components. Given that during training (a) participants’ responding matched the content of the rules, (b) this matching was observed throughout the whole phase, (c) the responses were almost exclusive to the instructed alternative, (d) response counts in each of the components were the same, (e) and this pattern of responding was maintained even when no reinforcement was provided, the results suggest that participants’ behavior was mainly under the control of the rules (Catania et al., 1982; Hayes et al., 1989).

However, the patterns of responding instructed by the rules were reinforced during the first phase. Therefore, it is also possible that the observed differential responding may have been exclusively owing to the programmed contingencies without any influence of the rules provided. For this reason, the rule-test phase was programmed. During the rule-test phase, all participants correctly recalled both rules when requested on their first try. This result provided more evidence of the control exerted by the rules.

In the critical part of the experiment, three outcomes were observed. First, participants exclusively responded to the instructed alternative when the context clearly signaled the opportunity to follow one and only one of the two rules (Rich and Lean components), replicating the results observed during the first phase. Second, when the context indicated that both rules could be followed (BS component), 6 out of 8 participants globally preferred the alternative that was instructed by the rule with the highest reinforcer rate. Third, when the context did not specify which rule had to be followed (NS component), preference for any of the alternatives was non-systematic, between-subject variability was the highest and participants favored either of the two alternatives, suggesting stimulus control as the main determiner of participants’ preferences.

Although the present results seem to support the hypothesis outlined in this study, a critical assumption of this hypothesis is that our results are due to participants discriminating between the two programmed reinforcer rates in the components, even when (a) their responding immediately conformed to what was stated by the two rules during the training phase with no differences in responding between components, and (b) their responding was about the same for the two VI alternatives during training, even when there were differences in reinforcement rate between the components. Therefore, a critical step is to demonstrate sensitivity to reinforcement under the present procedure without providing rules. If differential responding in each of the components is not demonstrated in the absence of performance rules, then this could imply that the programmed contingencies were, in fact, not discriminable, putting at odds the role of reinforcer rate in the experimental results. On the other hand, observing the development of orderly changes in the behavior of individual participants as a function of the programmed contingencies will support the role of rate of reinforcement as the main controlling variable, even when rules are involved. Additionally, qualitative differences in participants’ responding between experiments should be observed, since in the absence of rules, behavior should adapt more slowly to the programmed contingencies. Importantly, the results in the choice-test phase should be similar to the ones obtained in Experiment 1 since our underlying assumption is that the variable controlling behavior in choice situations, both when behavior is rule-governed or contingency shaped, is rate of reinforcement. Therefore, the aim of Experiment 2 was to test the effects of reinforcer rate under the present procedure without providing any performance rules.

## Experiment 2

### Method

#### Participants, Apparatus, and Setting

Eight additional undergraduate students (four women and four men) between 18 and 19 years old participated. The apparatus and setting were the same as in Experiment 1.

#### Procedure

The procedure was the same as in Experiment 1 with two exceptions. First, instructions were modified by removing the rules provided in Experiment 1.

Instructions were:

Welcome to “find the treasure.”

You are going to see two treasure chests on the screen. One on the left and one on the right. You must click on them to try to open them and get money. Each time you open a treasure chest, you will receive 0.7 cents of a peso (0.05 US dollars).

At the end of the experiment, the amount of money you earned will appear on the screen. If you do not have any doubts, click on the button “Continue.”

Second, the rule-test phase was removed, so participants only experienced the same training and choice-test phases of Experiment 1. These phases were the same as in Experiment 1. To advance from the reinforcement phase to the probe phase, 80% or more of participants’ responses had to be emitted to the VI alternative in both components for the last six observation periods. As in Experiment 1, at the end of the session the computer program showed the participant how much money he/she had earned. Participants received an average of 90 pesos (5 US dollars) at the end of the session.

#### Dependent Variables

For the reinforcement phase, the percentage of responses emitted to the VI alternative was the main dependent variable. As in Experiment 1, the mean response counts for each participant during the last six observation periods to each alternative were also reported. In the probe phase, responses to the different alternatives were counted. Then, these responses were divided by the total number of responses and multiplied by 100 to obtain the percentage of responses to each of the alternatives.

### Results

**Fig. 4:**
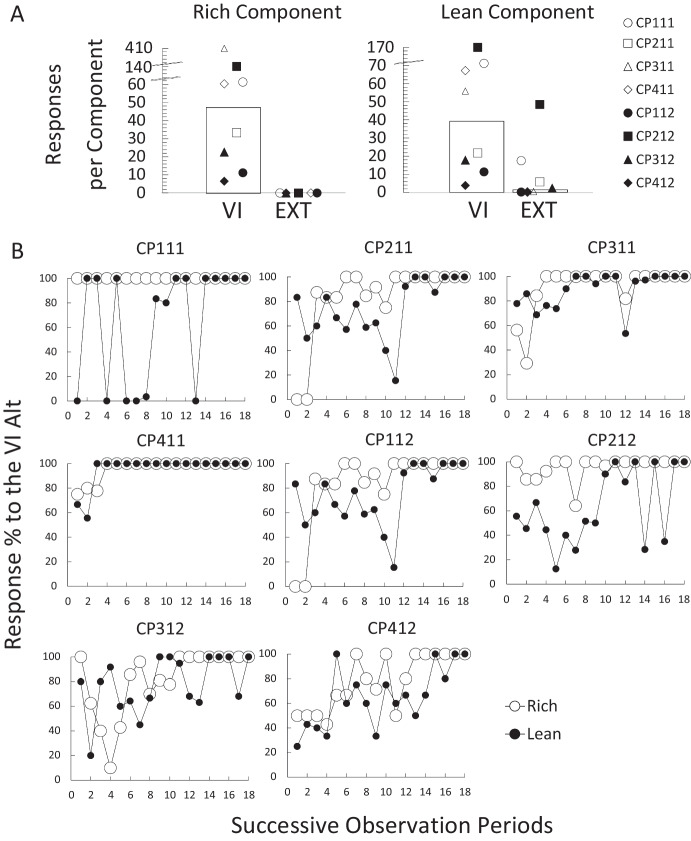
Choice analyses for the training phase in Experiment 2. *Note*: Panel A. Response counts for each alternative in each of the components. Each data point represents the estimated response count for each participant in each of the alternatives, calculated as the mean data from the last six observation periods. Bars represent the group median. VI (Variable-interval alternative) and EXT (Extinction alternative). Panel B. Percentage of responses to the variable-interval alternative as a function of successive observation periods

Figure 4 section A shows the mean response counts per alternative for each participant in the last six observation periods of the training phase. In the Rich component, all participants responded exclusively to the VI alternative (*T* 416.8, *M*_*dif*_ 52.1, *p* < 0.01; VI alternative 1st quartile 19.79*, Mdn* 46.83*,* 3rd quartile 81.37). A similar pattern was observed in the Lean component. All participants responded more to the VI alternative (*T* 344.1, *M*_*dif*_ 43.02, *p* < 0.01; VI Alternative 1st quartile 16.20*, Mdn* 38.83*,* 3rd quartile 60.16). However, in this component there were some participants who responded to the EXT alternative (CP212 48.5, CP111 17.5, CP211 5.83, and CP312 2.33; 1st quartile 0.29*, Mdn* 1.41*,* 3rd quartile 8.75).

**Fig. 5:**
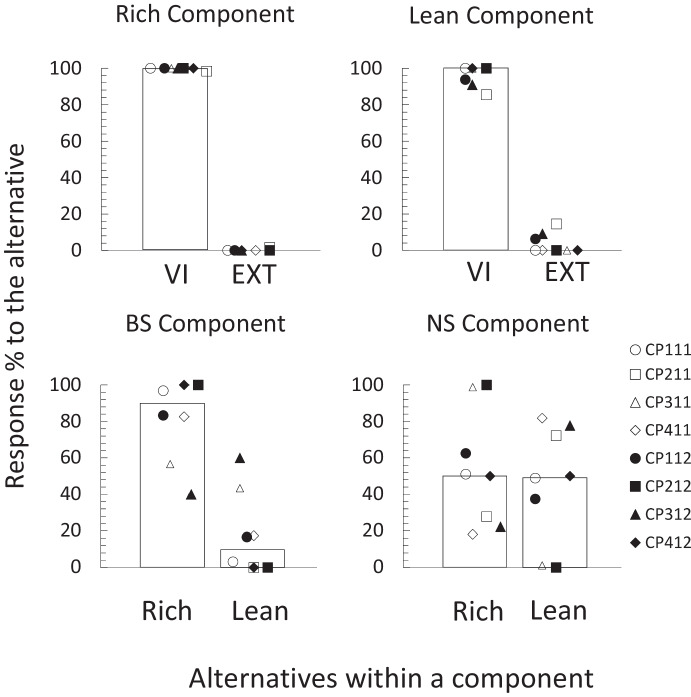
Choice analyses for the choice-test phase in Experiment 2. *Note*. Percentage of responses to each alternative in each of the components of the test phase in Experiment 2. Bars represent the group median: VI (Variable-Interval), Ext (Extinction), Rich (Rich-component associated alternative), Lean (Lean-component associated alternative)

Figure 4 section B shows the percentage of responses to the VI alternative as a function of all successive observation periods for all participants. Empty circles correspond to the Rich component and filled circles to the Lean component. Two different patterns of responding were identified. The first pattern corresponded to a gradual increase in the responses emitted to the VI alternative as observation periods passed. This pattern was observed for most participants (CP211, CP311, CP112, CP212, CP312, CP412). Although preference for the VI alternative gradually developed for both components, preference developed more rapidly for the Rich component only for participants CP112 and CP212. The second pattern of responding was an immediate and exclusive preference toward one or both VI alternatives according to the component in operation earlier in training. Participant CP111, for example, responded exclusively to the VI alternative in the Rich component, and alternated between options during the Lean component as observation periods passed. As the training ended, he responded exclusively to the VI alternatives for both components. On the other hand, participant CP411 exclusively responded to the VI alternatives in both components at an earlier stage of training and continued that way until the end of training,

Figure 5 shows the percentage of responses per alternative in each of the components in the choice-test phase. During the Rich component, all participants responded exclusively to the VI alternative (*T* 796.7, *M*_*dif*_ 99.5, *p* < 0.01). During the Lean component responding still favored the VI alternative (*T* 740.2, *M*_*dif*_ 92.5, *p* < 0.01), but some responses were observed for the EXT alternative. While responding to the VI alternative was nearly exclusive for most participants in this component (1st quartile 93%*, Mdn*100%*,* 3rd quartile 100%), participants CP211 (14.5%), CP112 (6.3%), and CP312 (9.1%) sampled the EXT alternative.

The test components showed similar results to Experiment 1. During the BS component most participants preferred the alternative they responded to during the Rich component (*T* 518.8, *M*_*dif*_ 64.8, *p* = 0.02; Rich 1st quartile 76.1%*, Mdn* 90.1%*, Q3* 100%; Lean 1st quartile 0%*, Md* 9.9%*,* 3rd quartile 23.89%). The only exception was participant CP312, who responded more to the opposite alternative (60%). In contrast, individual preferences were again unsystematic in the NS component (*T* 61.15, *M*_*dif*_ 7.64, *p* = 0.75; Rich 1st quartile 26.4%*, Mdn* 50.5%*,* 3rd quartile 71.58%; Lean 1st quartile 28.4%*, Mdn* 49.5%*,* 3rd quartile 73.61%). This time participants CP311 (Rich 98.8 %), CP112 (Rich 62.5%), and CP212 (Rich 100 %) favored the alternative instructed by the Rich rule, while participants CP211 (Rich 27.8%), CP411 (Rich 18.2 %), and CP312 (Rich 22.2%) favored the lean alternative. Participants CP412 (Rich 50%) and CP111 (Rich 51.1%) were indifferent.

### Discussion

Results showed that participants favored the alternatives associated with a higher reinforcer rate. When no rules were provided, two patterns of responding were observed during training: (a) participants gradually favoring the VI alternative in both components and (b) participants immediately developing a preference for one or both VI alternatives. The first pattern was the one displayed by most of the participants (6 of 8) and is consistent with research showing that preference for the alternative correlated with the highest relative reinforcer rate is observed when concurrent schedules are programmed in each of the components of a multiple schedule (Lobb & Davison, 1977; Pliskoff et al., 1968). The second pattern of responding also conforms to this statement. However, the two participants behaving in this way (CP111 and CP411) developed a rapid and exclusive preference for one or both VI alternatives, making their performances more similar to the ones observed in Experiment 1.

In the probe phase, preference for the VI alternative remained for both the Rich and Lean components. However, when both discriminative stimuli were present, preference favored the alternative associated with the greatest reinforcer rate (the one trained in the Rich component). Lastly, when there were no discriminative stimuli, unsystematic preferences were observed. The results obtained for the BS component conform with research showing that, when experimental participants are presented with a choice between multiple stimuli, each previously associated with reinforcement, they tend to select the alternative associated with the highest reinforcer rate even when this choice does not correspond to the trained relations (Dube & McIlvane, 1997; Hartl & Fantino, 1996). Furthermore, the results bear some resemblance with a recent finding by Cowie et al. (2020), who showed that when conflicting discriminative information is provided in choice situations, preference favors the higher valued alternative.

The performance of two participants (CP111 and CP411) during the training phase were similar to the ones observed in Experiment 1. In this regard, a question worth asking is if their performances were influenced by self-rules? It has been observed that human participants produce and follow self-rules during operant tasks (Baumann et al., 2009; Rosenfarb et al., 1992; Santos et al., 2015). Therefore, it is possible that some, if not all, participants in this experiment were involved in rule production. In this experiment, the methodological arrangement did not provide opportunities to explore this possibility. However, the fact that most participants in Experiment 2 during training developed a preference for the reinforced alternative differently than the participants in Experiment 1 highlights the differential effect that rules provided by others have on individual behavior in comparison to performances produced by verbally competent humans in novel situations in the absence of externally provided rules (Baumann et al., 2009; Rosenfarb et al., 1992). Additionally, the fact that similar results in the choice-test phase were found in both experiments is in accordance with the idea that reinforcer rate is the ultimate controlling variable in rule-governed or contingency shaped choice situations. Given that the aim of this experiment was to demonstrate that participants were sensitive to the programmed contingencies in the sense that manipulations had to affect, “behavior in an orderly and replicable manner” (Madden et al., 1998, p 7), the obtained results support the objective of this experiment.

## General Discussion

The aim of this research was to model a situation where the effect of two conflicting rules, each associated with different reinforcer rates, could be tested in a situation where choice was induced between the rules. The main hypothesis of this study was that when two conflicting rules can be followed in a given situation, the rule associated with the highest reinforcer rate will be followed. The results of both experiments appear to support the predictions derived from the main hypothesis of the study. However, the experimenter believes that there is a methodological aspect of the study that makes it difficult to interpret the results, specifically when explaining the results of the choice-test phase.

In this phase, both experiments showed the same general results: when both discriminative stimuli were presented, there was a preference for the alternative that provided more reinforcement, while indifference was observed in the absence of discriminative stimuli. Although the main hypothesis of the study predicted this outcome in both experiments, the critical question is: how do we know that the rules provided to participants were functionally related to the outcome observed *during the choice-test phase* in Experiment 1? In other words, how do we know if participants were, in fact, choosing which rule to follow and not behaving in another way? The problem is that no further tests for rule-following were conducted during this phase. Therefore, there is no certainty that rule-following behavior, and therefore the choice process hypothesized in this study, was occurring in Experiment 1. In fact, given that the same results were observed in the absence of rules, an alternative explanation for the findings of Experiment 1 could be that contingencies of reinforcement were the main determinants of participants’ performance while rules were partially or not related at all to the behavioral outcome observed in the last phase.

It is important to consider that the main hypothesis of this study also sees reinforcer rate as the ultimate controlling variable. However, the crucial difference with the previous explanation is that our hypothesis sees the behavioral control of the rules provided to participants at the beginning of Experiment 1 as the main factor determining participants’ performance on the choice-test phase. In other words, it assumes that rules were controlling participants’ behavior the whole experiment and that the individual effect of each rule was determined by their associated reinforcer rate. Under this view, the results of the experiment could be interpreted in a different way: during the training phase in Experiment 1, the rules provided controlled their behavior since the beginning of the experiment, likely because of the participants’ shared history of reinforcement associated with following rules in the past (i.e., tracking; see Hayes et al., 1989). Once participants interacted with the task and followed the rules, their behavior was reinforced, experiencing a correspondence between what was stated by the rules and the way contingencies were programmed. This might have caused persistent rule-following throughout the whole phase, as the patterns of responding seem to suggest. This outcome seems likely if one also considers that sampling to the uninstructed alternative did occur but was infrequent and transient. Once participants interacted with the rule-test phase, they answered correctly the four test sentences on the first try (all participants did). So, at the end of the experiment, given that (a) verbal recall of the rules was demonstrated, (b) patterns of responding suggested rule-following, and (c) differential reinforcement was provided for following the two rules once a choice was induced between the rules (BS Condition), participants could have chosen which rule to favor based on their past experience (the reinforcer rate associated with following each of the rules) as the patterns of responding in the last phase seem to suggest.

Nevertheless, considering the methodological difficulty previously outlined, there is currently no way to know which of the two possible explanations describes more accurately the results of Experiment 1. While data are compatible with the main hypothesis of the study, the issue is that an additional test is needed to confirm the degree of behavioral control exerted by the rules on participants’ choices during the choice-test phase in Experiment 1. A first way to solve this issue might be to extend the presentations of the BS component during the choice-test phase. An important aspect of this component was that extinction was programmed to both alternatives. Given that it has been observed that instructional content extends resistance to change (Podlesnik & Chase, 2006), extending the presentations of the BS component under extinction might reveal differences in participants’ behavior when rules are provided, as compared to situations where rules are omitted. In addition to conducting interviews after the experiment, a protocol involving talk-aloud procedures (Cabello et al., 2004; Hayes, 1986; Miguel, 2018) might be used to further study the validity of the proposed behavioral process. This could shed some light on whether or not participants do in fact get involved in a process where the rules previously given are chosen in the choice-test phase or if another behavior is taking place. Together, this set of methodological improvements could allow for a stronger test of the main hypothesis of the study in future experiments.

Another important aspect is the presentation modality in this experiment. Although similar operant studies using online procedures, both using discrete trial (Colbert et al., 2019; Ruiz et al., 2022) or free operant procedures (Robinson & Kelley, 2020), have successfully produced orderly data, using “remote participants” is not yet a common practice in the experimental analysis of human behavior (Ritchey et al., 2021). The results described in this report seem to support the usefulness of such procedures in producing orderly human data. However, there might be subtle differences between the different online procedures cited above and this experiment (such as the presence of the experimenter and the pre-assessment procedures) that might be important during experimentation. Therefore, future experiments should aim to replicate the present results in laboratory settings to further validate the experimental data presented in this report.

From a theoretical point of view, the results of this study seek to stimulate research related to rule-following and culture. In the context of human culture, for example, rules are forms of behavior used by the members of a culture to regulate the behavior of others within the group. Baum (1995) explains this process by distinguishing between proximate and ultimate contingencies. According to Baum, when a person is given a rule and decides to follow it, his/her initial behavior is under the control of the rule (rule-governed behavior). This rule-following behavior, in turn, is maintained by the immediate social reinforcement provided by the verbal community (proximate contingency). If the same person continues to behave as instructed by the rule, eventually his/her behavior will contact the consequences associated with behaving this way, being that these consequences are now the main controllers of behavior (ultimate contingency). It is in this way that the members of a culture, and the various rules they provide and enforce, help shape an individual’s behavior in accordance with the cultural practices that have been selected throughout that group’s history.

From the perspective of this study, it is recognized that following a rule is just one of many different responses that an individual might display in a particular situation. If all behavior is choice (Baum, 2010), this means that when a person follows one rule instead of another, his/her behavior is a form of choice, a verbal choice, controlled by the distribution of reinforcement associated with following both the rules. Therefore, this type of choice could also be described by the matching law (Baum, 1974, 2018). Based on Baum’s view, a possibility is that this distribution of reinforcement might be determined by the differential consequences associated with following each rule, arranged by the members of a verbal community in the context of cultural practices. If true, the individuals who provide the most effective consequences could have an important role in determining the tendency of a person to follow or not follow rules as well as to which rules are followed and from whom. In fact, this view might turn our attention to *who* is providing the rule and how the individual effect of different individuals providing different rules is determined on the behavior of the listener. Seeing rule-following behavior as choice could also change the logic behind some interventions that target rule-following: the analyst could conceptually model or find a way to empirically estimate the frequency of reinforcement associated with following a rule in relation to the reinforcement associated with incompatible behaviors and then manipulate the frequency of reinforcement of one of the elements of the interaction in order to change the levels of the target behavior in a desired direction (see McDowell, 1988, p.105).

In summary, the experiments described in this report are concerned with how our behavior transforms as a function of the many rules we are given by the members of our verbal communities. Given that some of those rules might instruct different or even contradictory courses of action, the study of rule-following behavior in the context of choice might allow us to understand better the factors that determine the differential influence of a rule in the context of other rules. This is a valuable endeavor in that it might shed some light on the verbal dimension behind our daily choices while further extending the principles of behavior developed in classic choice research to the area of verbal behavior. This procedure and the proposed methodological improvements could be the starting point for new research focusing on the study of complex decision making where the verbal component is central.

## Data Availability

The datasets generated during the current study are available from the corresponding author on reasonable request.
